# Prognostic Impact of Melatonin Receptors MT1 and MT2 in Non-Small Cell Lung Cancer (NSCLC)

**DOI:** 10.3390/cancers11071001

**Published:** 2019-07-17

**Authors:** Karolina Jablonska, Katarzyna Nowinska, Aleksandra Piotrowska, Aleksandra Partynska, Ewa Katnik, Konrad Pawelczyk, Alicja Kmiecik, Natalia Glatzel-Plucinska, Marzenna Podhorska-Okolow, Piotr Dziegiel

**Affiliations:** 1 Division of Histology and Embryology, Department of Human Morphology and Embryology, Wroclaw Medical University, 50-368 Wroclaw, Poland; 2 Department of Thoracic Surgery, Wroclaw Medical University, 53-439 Wroclaw, Poland; 3 Department of Thoracic Surgery, Lower Silesian Centre of Lung Diseases, 53-439 Wroclaw, Poland; 4 Division of Ultrastructure Research, Wroclaw Medical University, 50-368 Wroclaw, Poland; 5 Department of Physiotherapy, University School of Physical Education, 51-612 Wroclaw, Poland

**Keywords:** melatonin, melatonin receptor, MT1, MT2, non-small cell lung carcinoma, immunohistochemistry, cancer

## Abstract

*Background*: Several studies have investigated the inhibitory effect of melatonin on lung cancer cells. There are no data available on the prognostic impact of melatonin receptors MT1 and MT2 in non-small cell lung cancer (NSCLC). *Materials and Methods*: Immunohistochemical studies of MT1 and MT2 were conducted on NSCLC (N = 786) and non-malignant lung tissue (NMLT) (N = 120) using tissue microarrays. Molecular studies were performed on frozen fragments of NSCLC (N = 62; real time PCR), NMLT (N = 24) and lung cancer cell lines NCI-H1703, A549 and IMR-90 (real time PCR, western blot). *Results*: The expression of both receptors was higher in NSCLC than in NMLT. Higher MT1 and MT2 expression levels (at protein and mRNA) were noted in squamous cell carcinomas (SCC) compared to adenocarcinomas (AC). MT1 immunoexpression decreased as both the tumour size and the cancer stage increased in the whole cohort, while MT2 decreased as the cancer stage increased, with lymph node involvement (in the whole study group) and increasing malignancy grade (in SCC). Higher expression of MT2 was associated with a favorable prognosis. MT2 was an independent prognostic factor for overall survival (OS) in all analyzed NSCLC and in smoking patients. *Conclusions*: Our observations may point to the potential prognostic significance of MT2 in NSCLC.

## 1. Introduction

Lung cancer is one the most frequently diagnosed types of cancer and the leading cause of cancer-related deaths. According to the World Health Organization (WHO) statistics, in 2018 there were over two million cases of lung cancer worldwide, accounting for 11.6% of all cancers [1]. There are two main types of lung cancer: small cell lung cancer (SCLC) and non-small cell lung cancer (NSCLC) [2]. Small cell lung cancer (highly malignant and mostly caused by smoking) accounts for about 15% of all cases [2]. Non-small cell lung cancer (85% of cases) includes three histological types: adenocarcinoma (AC) (40%, most often diagnosed in women and non-smokers), squamous cell carcinoma (SCC) (30%, most commonly found in smoking men) and large cell carcinoma (15%, rapid growth and spread) [2,3]. Thyroid transcription factor-1 (TTF-1), a member of the Nkx2 family of transcription factors, is used for sub-classification of adenocarcinomas with ~80% sensitivity, while p63 is a marker for identification of squamous differentiation [4]. The main causes of lung cancer are long-term smoking, occupational factors (exposure to beryllium, chromium, nickel, asbestos, benzene, ether, aromatic hydrocarbons and to radiation) and exposure to indoor and outdoor air pollution [5,6]. A significant, though often overlooked problem, is related to non-smoking patients, in whom the development of lung cancer is not the result of cigarette smoking, but most likely a mutation in the genes [7]. Epidemiological studies have shown also that impaired secretion of melatonin (for example caused by night-shift jobs), increases the risk of lung cancer [8]. In addition, patients with NSCLC were characterized by decreased synthesis and metabolism of melatonin [9]. Active smoking, the main cause of lung cancer, also reduces blood melatonin level [10].

Lung cancer is predominantly diagnosed at an advanced stage of development and surgery remains the mainstay of treatment. Adjuvant treatment, such as chemotherapy or radiation, has a negative effect on normal cells. It is also associated with a high risk of complications and the effectiveness is significantly limited [11]. Numerous studies have been conducted in order to improve standard therapies by using a combination of routine chemotherapeutics with substances such as melatonin (5-methoxy N-acetyltryptamine) [12].

In vertebrates, melatonin is mainly synthesized in pinealocytes of the pineal gland and its secretion is synchronized with the circadian cycle [13]. Melatonin is a compound with pleiotropic effect and is reported to be a substance that delays the aging process, stimulates the immune system and has anti-cancer activity [13,14,15]. Melatonin can act either directly or through the membrane melatonin receptors (MT1, MT2). The influence of this hormone on tumor development may be the result of its antioxidant properties and the inhibitory effect on calmodulin or the mitogen-activated protein kinase/extracellular signal-regulated kinase (MEK/ERK) signaling cascade with the participation of the melatonin receptors [16,17,18]. 

MT1 and MT2 bind melatonin with high affinity (20–200 pM) [19]. Both receptors belong to the G-Protein Coupled Receptors (GPCR) family and are composed of seven transmembrane regions with a helical structure [20]. Human melatonin receptors are 350 (MT1) and 362 (MT2) amino acids (aa) long, with molecular weights of 39–40 kDa and 55% aa homology overall and 70% within membrane domains [19,20]. The chromosomal locations of melatonin receptors genes are 4q35.1 for *MTNR1A* and 11q21–q22 for *MTNR1B* [20,21]. Melatonin through its receptors inhibits the activity of adenylate cyclase, thereby lowering the intracellular level of cyclic adenosine monophosphate (cAMP). As a result, melatonin regulates the activity of protein kinase C (PKC), protein kinase A (PKA) or mitogen-activated protein kinases (MAPKs). It has an impact on the phosphorylation level of transcription factors, such as CREB (cAMP response element-binding), c-jun, c-fos, c-myc and downregulation of genes associated with the process of proliferation [20,21,22].

So far, our studies have been focused on the assessment of the role of melatonin receptors in melanoma [23], breast cancer [24] and ovarian cancer [25,26]. Based on the obtained results, we showed that a higher expression of MT1 in breast cancer was an independent prognostic factor for longer overall survival (OS) and event-free survival (EFS) in the group of estrogen receptor positive (ER+) tumors [24]. Currently, there are no reports describing the predictive and prognostic impact of membrane melatonin receptors MT1 and MT2 in NSCLC. 

Clinical studies show that the use of melatonin increases the effectiveness of chemotherapy, reduces chemotherapy-related adverse effects, prolongs survival and improves the quality of life of patients with NSCLC [27,28]. Numerous studies demonstrate the sensitizing effects of melatonin in combination with conventional chemotherapeutic agents, such as cisplatin or doxorubicin [29,30,31,32]. It has been shown experimentally that melatonin reduced carcinogenesis of urethane-induced tumors in the murine lung [33]. Additionally, melatonin, through its antioxidant activity, inhibits oxidative stress induced by smoking and protects lungs against damage [34]. In experiments with lung cancer cell lines, melatonin reduced cell viability (lines A549 and PC9), decreased the ability of cells to metastasize (line A549) and increased cell apoptosis [35]. Furthermore, Yun et al. suggested the potential use of melatonin and gefitinib in NSCLC patients with epidermal growth factor receptor (EGFR) mutations (T790M) resistant to tyrosine kinase inhibitors [36]. Disruption of EGFR function is one of the most important elements in the pathogenesis of lung cancer. The functions of this receptor may be impaired by various mechanisms, such as a defective mechanism of inhibition of receptor activity, increased ligand concentration, EGFR self-stimulation via autocrine switch mechanism, heterodimerization, cross-talk signal exchange and cross-talk phosphorylation [37].

In the future, melatonin receptors may become a factor indicating the possibility of using melatonin in complementary therapy of NSCLC. The aim of the study was to assess the expression of melatonin receptors at both the protein and mRNA levels and the associations of the results obtained with the selected clinical and pathological factors in NSCLC. This may increase the chance to personalize anti-cancer therapy. Choosing the correct course of treatment based on the individual genetic array reduces the risk of side effects of treatment, increases the survival rate of patients and helps to avoid additional costs associated with improper therapy.

## 2. Results

### 2.1. Immunoexpression of MT1 and MT2 in Relation to Clinico-Pathological Parameters of the Patients 

#### 2.1.1. MT1 Melatonin Receptor

**Figure 1:**
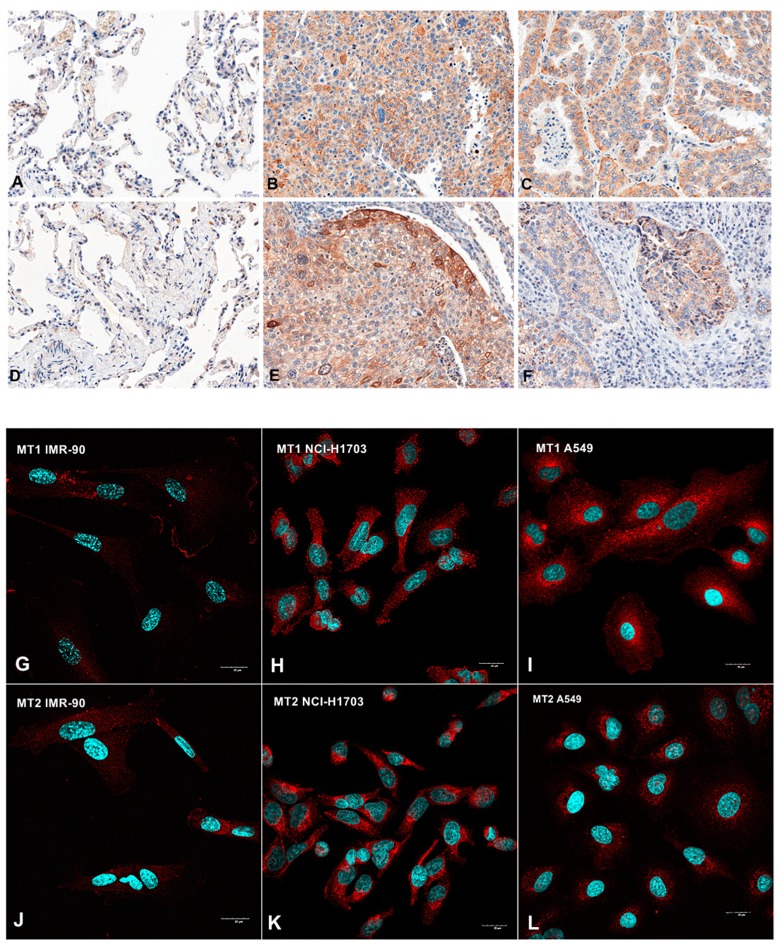
Immunohistochemical (**A**–**F**) and confocal (**G**–**L**) images showing membranous/cytoplasmic expression of melatonin receptors MT1 (**A**–**C**; **G**–**I**) and MT2 (**D**–**F**; **J**–**L**) in non-malignant lung tissue (NMLT) (**A**,**D**) and human lung fibroblast (**G**,**J**), cancer cells of squamous cell carcinomas (**B**,**E**,**H**,**K**) and adenocarcinomas (**C**,**F**,**I**,**L**). Magnification ×200 (**A**–**F**) and ×600 (**G**–**L**).

**Figure 2:**
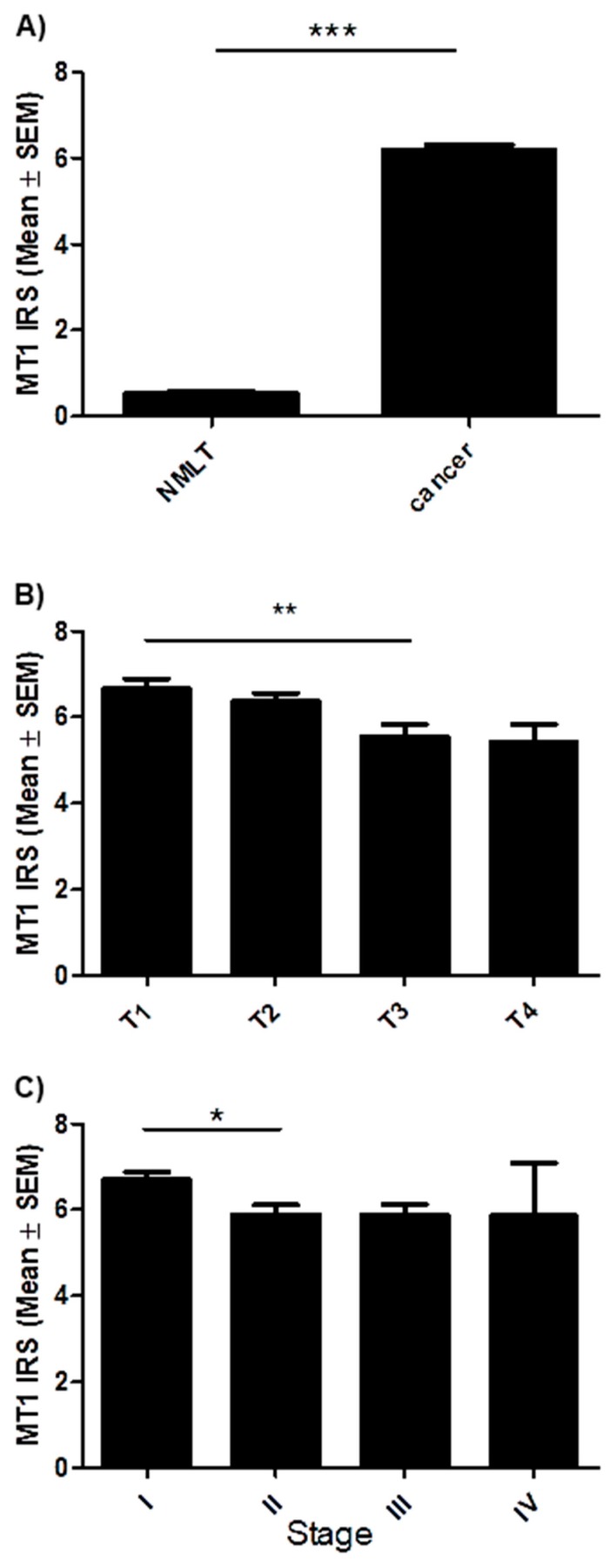
Expression level of melatonin receptor MT1 in regard to patients clinico-pathological factors. (**A**) Comparison of immunohistochemical MT1 expression in non-malignant lung tissue (NMLT) with its expression in non-small cell lung carcinoma (*** *p* < 0.001); Mann Whitney test. Expression levels of MT1 in respect to (**B**) tumor size (** *p* < 0.01) and (**C**) clinical cancer stage (* *p* < 0.05); Dunn’s Multiple Comparison test.

Positive membranous/cytoplasmic expression of MT1 was noted in 758 specimens (96.4%) (Figure 1). The mean value of MT1 expression (IHC) was 6.2 ± 3.4. The whole scores were divided into two groups: a score of 0–6 pts was considered ‘low’ (420), while a 7–12 pts one was considered ‘high’ (366) (Table 1). In all studied cases a significantly higher expression was noted in cancer than in non-malignant lung tissue (NMLT) (*** *p* < 0.001; Mann Whitney test, Figure 2A). The Kruskal-Wallis test demonstrated a decreasing level of MT1 (immunohistochemistry, IHC) with increasing tumor size (in total; P = 0.0028). A significant difference was noted between T1 and T3 (** *p* < 0.01, Figure 2B). MT1 expression level decreased with increasing cancer stage (in the whole cohort; *p* = 0.0181); a significant difference was noted between stages I and II (* *p* < 0.05, Figure 2C). MT1 receptor showed a low positive (r ≤ 2; *p* < 0.05) correlation with MT2 (in the whole cohort, AC, SCC), p63 (in the whole cohort and AC), TTF-1 (in AC) and EGFR (in the whole study group, AC, SCC). There were no significant relationships (in the whole study group, SCC, AC) between MT1 and gender, tumor grade (G), lymph node status (N), metastases (M), smoking status, living in the city, distal metastases.

#### 2.1.2. MT2 Melatonin Receptor

**Figure 3:**
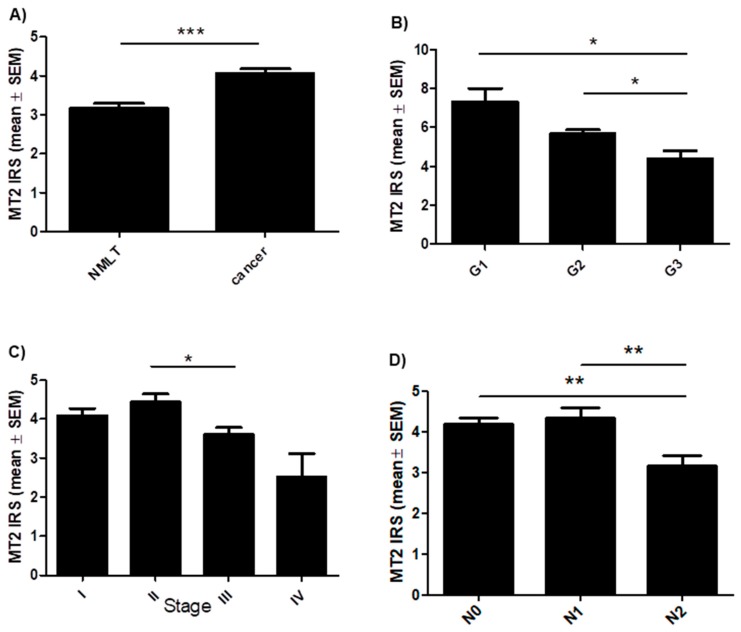
Expression level of melatonin receptor MT2 in regard to patients clinico-pathological factors. (**A**) Comparison of immunohistochemical MT2 expression in non-malignant lung tissue (NMLT) with its expression in non-small cell lung carcinoma (*** *p* < 0.001); Mann Whitney test. Expression levels of MT2 in respect to (**B**) malignancy grade (G) of the tumor in squamous cell carcinoma (SCC) (** *p* < 0.01), (**C**) clinical cancer stage (in total) (* *p* < 0.05) and (**D**) the presence of lymph node metastases (in total) (** *p* < 0.01); Dunn’s Multiple Comparison test.

A membranous/cytoplasmic expression of MT2 was noted in 680 cases of NSCLC (86.5%) (Figure 1). The mean value of MT2 expression (IHC) was 4.1 ± 3.1. Sections with a score of 0–3 pts were considered ‘low’ (390) while those with 4–12 pts, ‘high’ (396) (Table 1). A significantly higher level of MT2 was observed in NSCLC than in NMLT (*** *p* < 0.001) (Figure 3A). The Kruskal-Wallis test demonstrated a decreasing level of MT2 (IHC) with increasing malignancy grade (G) of the tumor in SCC (*p* = 0.0034). A significant difference was noted between G1 and G3; G2 and G3 (* *p* < 0.05); Figure 3B. MT2 expression decreased as the cancer stage increased (in the whole cohort; *p* = 0.0015; Kruskal-Wallis test); a significant difference was noted between stages II and III (* *p* < 0.05); Figure 3C. We also noted a decreasing level of MT2 with increasing degree of spread to regional lymph nodes (N) (in the whole cohort; p = 0.0023; Kruskal-Wallis test); Figure 3D. Statistically significant differences were also seen between N0 vs N2 and N1 vs N2 (** *p* < 0.01). The expression of this receptor was significantly higher in smoking patients (*p* = 0.0039). Furthermore, we observed a low (r ≤ 0.2) positive significant (*p* < 0.05) correlation of MT2 with: MT1 (in the whole cohort, AC, SCC, p63 (in AC and SCC), EGFR (in the whole study group, SCC); a moderate positive (r = 0.4) correlation with: p63 (in total); low negative (r = −0.24) with TTF-1 (in total). No other significant associations were noted (in the whole cohort, AC, SCC) between MT2 and gender, metastases (M) and living in the city.

### 2.2. Relations between MT1, MT2 (Protein and mRNA) Expression and Histology Subtype

**Figure 4:**
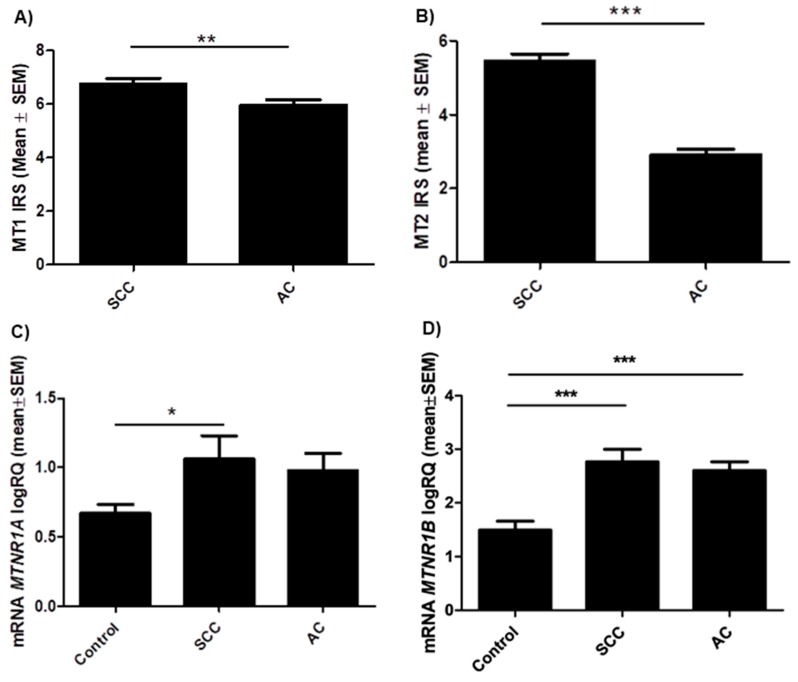
Protein and mRNA expression levels of melatonin receptors in the tissue of particular non-small cell lung carcinoma (NSCLC) subtypes: squamous cell carcinoma (SCC) and adenocarcinoma (AC). Immunohistochemical expression levels of melatonin receptors (**A**) MT1 (**B**) MT2 (** *p* < 0.01); Mann Whitney test. Relative quantification (RQ) mRNA expression levels of (**C**) MTNR1A mRNA (* *p* < 0.05) and (**D**) MTNR1B mRNA in NSCLC in respect to histology type (*** *p* < 0.001); Dunn’s Multiple Comparison Test.

**Figure 5:**
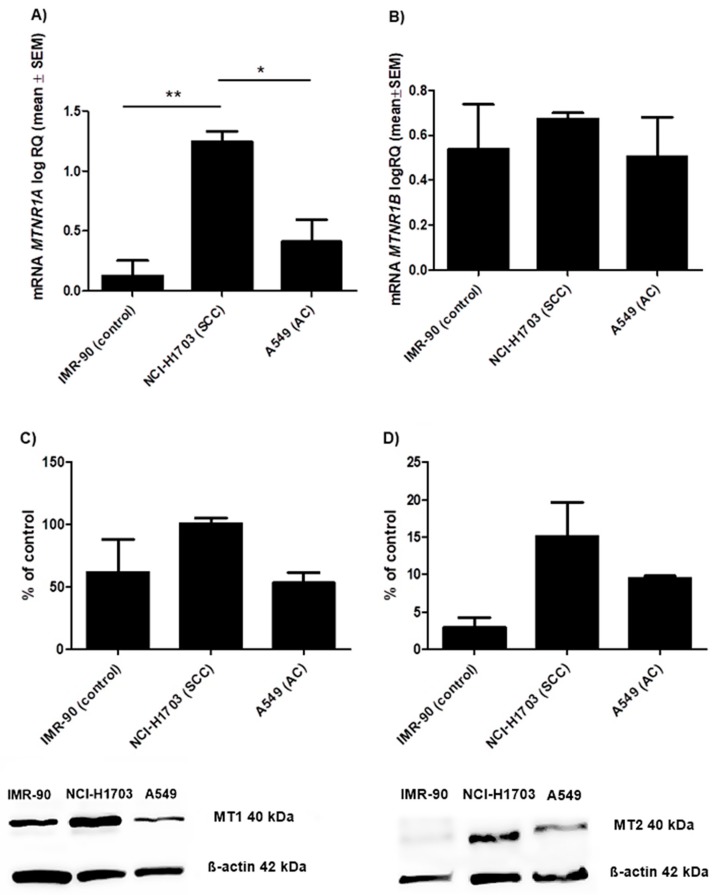
Expression level of melatonin receptors in lung cancer cell lines that represents different histology subtypes: NCI-H1703 (squamous cell carcinoma, SCC), A549 (adenocarcinama, AC) and IMR-90 (control). Relative quantification (RQ) mRNA expression levels of (**A**) MTNR1A mRNA (* *p* < 0.05; ** *p* < 0.01; Bonferroni Multiple Comparison test) and (**B**) MTNR1B mRNA. Western blot illustrates the presence of melatonin receptors (**C**) MT1 and (**D**) MT2 in the studied cell lines. Densitometric analysis of the bands demonstrates differences in MT1 and MT2 protein contents between the selected cell lines. All the experiments were performed in triplicates.

Immunohistochemical analysis demonstrated significantly higher expression levels of MT1 (*p* = 0.0022) and MT2 (*p* < 0.0001) in cases with SCC than with AC (Figure 4A,B). These results were confirmed by molecular studies of tissue samples (62 NSCLC) and lung cancer cell lines (NCI-703, A549 and IMR-90). The expression of *MTNR1A* and *MTNR1B* genes was found in all examined tissue samples. The product of the real-time PCR reaction was related to the reference gene β-actin. The obtained data showed statistically significant differences of relative quantification (RQ) MTNR1A mRNA (p = 0.022) (Figure 4C) and MTNR1B mRNA (*p* < 0.0001) (Figure 4D) expression levels according to the histology type of NSCLC. A significant difference at MTNR1A mRNA level was noted between the control and SCC (* *p* < 0.05) while in the case of MTNR1B mRNA significant differences were noted between the control and SCC as well as the control and AC (*** *p* < 0.001). Lung cancer cell lines that represent different histology subtypes showed a higher expression level of MTNR1A mRNA (*p* = 0.0058) (Figure 5A) in NCI-H1703 (SCC) cell line compared to A549 (AC) (* *p* < 0.05). MTNR1B mRNA expression levels in selected lung cancer cell lines (Figure 5B) were statistically insignificant.

The protein levels of MT1 and MT2 were measured by western blot. Anti-MT1 (Figure 5C) and anti-MT2 (Figure 5D) antibodies marked bands of 40 kDa. Densitometric analysis showed the differences in MT1 protein contents between cell lines differing in terms of histology subtype: NCI-H1703 (101%), A549 (53%) and IMR-90 (62%) as the control. The differences in MT2 protein contents were also observed between the above cell lines i.e., NCI-H1703 (15%), A549 (9.5%) and IMR-90 (3%). The results from the western blot are presented as the means for percentage values of MT1/β-actin and MT2/β-actin optical density ratios obtained from three independent experiments.

### 2.3. Survival Analysis

**Figure 6:**
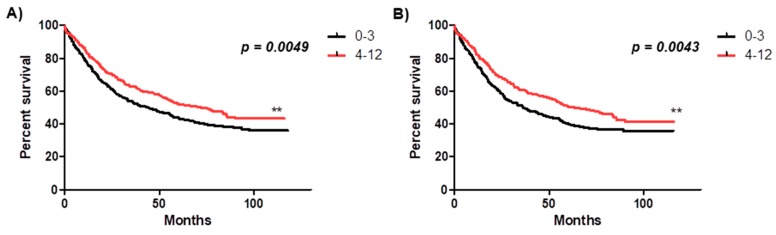
Kaplan-Meir survival curves reflecting the prognostic impact of MT2 melatonin receptor expression (immunohistochemistry, IHC) on overall survival (OS). Patients were followed up for 50 ± 34.45 months and grouped according to the median value of immunohistochemical expression of MT2 receptor in non-small cell lung cancer (NSCLC) tissues. Black solid curves represents patients with lower expression of MT2 receptor (0–3) while solid red curve represent patients with higher expression of MT2 receptor (4–12). (**A**) The overall survival rate in all studied patients was 44% for NSCLC patients with lower (0–3) expression of MT2 (N = 408) and 72% for NSCLC patients with higher (4–12) expression of MT2 (N = 378) (** *p* = 0.0049). (**B**) The overall survival rate in the group of smoking patients was 36% for NSCLC patients with lower (0–3) expression of MT2 (N = 332) and 63% for NSCLC patients with higher (4–12) expression of MT2 (N = 333) (** *p* = 0.0043).

The prognostic significance of melatonin receptor expression in NSCLC was analysed in relation to OS in the whole study cohort, both in the smoking and non-smoking patients groups in AC and SCC. A higher expression of MT2 was associated with a favorable prognosis in the whole cohort (** *p* = 0.0049) and among smoking patients (** *p* = 0.0043) (Figure 6). The expression level of melatonin receptors was not associated with OS of NSCLC patients with AC and SCC analysed separately.

Univariate analysis demonstrated that some clinico-pathological factors were significantly associated with a poorer OS. These factors were the following: male status, larger primary tumor size (T2–T4), lymph node metastasis, advanced stage (II–IV) (in the whole study cohort, in the group of non-smoking and smoking patients, in AC and SCC); age ≥60 years (whole cohort, smoking), higher G status (SCC); smoking status (whole cohort, AC); high expression of EGFR (AC). Additionally, high expression of p63 (in total) was associated with a favorable prognosis (Table 2).

Multivariate survival analysis was performed for all factors that were significantly associated with the OS in the univariate analysis. The Cox proportional hazards regression model revealed that MT2 was an independent prognostic indicator for OS in patients with NSCLC in the whole study cohort (Table 2A) and in the group of smokers (Table 2B). The multivariate analysis demonstrated that gender, age, tumor size and lymph node metastases were independent prognostic factors in the whole study cohort (Table 2A) and in the group of smoking patients (Table 2B). Additionally, the TNM stage was an independent prognostic factor in the whole study cohort (Table 2A) and in non-smoking patients (Table 2B), while p63 expression was an independent prognostic factors in the whole study cohort (Table 2A). Expression of melatonin receptors was not associated with OS in the group of non-smoking patients (Table 2B).

Multivariate analysis showed that melatonin receptors were not associated with OS in the groups of AC and SCC analysed separately. In the AC subgroup multivariate analysis demonstrated that male status, tumor size, lymph node metastases and the expression of EGFR were independent prognostic indicators for OS. In the SCC subgroup only tumor size was found to be an independent prognostic marker of OS (Table 2C). 

## 3. Discussion

Non-small cell lung cancer (NCSLC) is a type of cancer that is usually diagnosed at an advanced stage of the disease. As a result, surgery, chemo- and radiotherapy are the main treatment methods available to prolong the survival of NSCLC patients. In recent years many studies have suggested that the drug synergy of melatonin with standard adjuvant therapy may enhance the efficiency of the treatment, reduce the side effects of chemotherapy and may improve the quality of life in NSCLC patients [27,28,38]. In addition, scientific evidence supports the role of melatonin receptors in oncostatic effects of melatonin in different types of cancer (breast, ovarian, prostate, melanoma) [23,24,25,26,39]. 

So far, it has been shown that melatonin exerts antiproliferative effects and induces apoptosis in lung cancer cell lines, especially in NSCLC [29,30,40,41]. Several studies have provided evidence for the antioxidative and free radical scavenging effects of melatonin. However, receptor-mediated mechanism, especially in lung cancer cells still remains unknown [35]. Currently, there are no in vitro studies, especially with human tissue material, examining the importance of melatonin receptors in lung cancer cells. In this study, we analyzed for the first time the expression of melatonin receptors MT1 and MT2 in a large series of NSCLC cases and showed the relationships between protein level, gene expression and clinico-pathological features using IHC and the real-time PCR techniques.

A positive reaction for both analysed melatonin receptors was identified in approximately 90% of NSCLC cases. The IHC results revealed a higher expression of both receptors in neoplastic changes in relation to healthy individuals. Similar relationships were for the first time demonstrated by Dillon et al. in immunohistochemical studies on MT1 receptor in human breast cancer specimens and were confirmed by our previous studies [24,42]. Nasrabadi N et al. also showed higher expression of MT1 and MT2 receptors in gastric adenocarcinoma compared to the control group [43,44]. Cancer cells have more genetic changes than normal cells. Some gene changes cause an increase production of a protein in comparison to normal cells. Due to the diminished concentrations of melatonin in peripheral tissues of NSCLC patients we suggest a compensatory and defending mechanisms leading to up-regulation of melatonin receptors [43]. Up-regulations of melatonin receptors in cancer cells lead to inhibition of some protein kinases (PKA, protein kinase A; PKC, protein kinase C; MAPK, mitogen-activated protein kinase), decrease the phosphorylation of transcription factors, i.e., CREB (cAMP response element-binding) and reduce the expression of genes associated with the process of proliferation [25,45].

Our recently published reports described an inverse relationship between MT1 and an increasing malignancy grade of the breast and ovarian cancers at the protein and mRNA levels [24,25,26]. In ovarian cell lines the highest level of MT1 receptor expression was observed in normal ovarian epithelial cells IOSE 364 while the lowest one was present in poorly differentiated ovarian cancer cells OVCAR-3. Interestingly, in lung cancer we noted a trend of down-regulations of MT2 with malignancy grade (G) in SCC types of analyzed tumors and no relation between MT1 and G. Recently, Nasrabadi et al. have shown the association between MT1 receptor mRNA levels and malignancy grade in patients over 50 years of age but they have not been able to observe any relationship between MT2 receptor expression and the clinico-pathological features of gastric tumor [43].

Moreover, an analysis of expression intensities of melatonin receptors (IHC) in relation to clinico-pathological parameters showed a significant decrease of both receptors with increasing clinical stages (TNM). Additionally, we noted a decreased level of MT1 protein with increasing tumor size (pT) and a decreased level of MT2 with lymph node metastases. This findings are in accordance with our previous results showing a lower expression of MT1 in triple negative breast tumors that showed higher malignancy and less favorable prognosis [24]. This fact suggests a variable level of melatonin receptors considering the TNM. Mazzoccoli et al. demonstrated that the melatonin level was lower in NSCLC patients and decreased with the stage of the tumor [46]. Melatonin through MT1 and MT2 receptors can have preventive role and reduce the risk of lung cancer in early stages of cancerogenesis [45]. 

Cigarette smoking is responsible for 90% of lung cancer incidence. In 2015, 55% of lung cancer deaths in women and over 70% of lung cancer deaths in men were due to smoking [47]. On the one hand, active smoking decreases human blood melatonin levels [10]. However, melatonin treatment has a reducing effect in cigarette smoke-induced lung diseases [41]. Several mechanisms explaining the inhibitory effects of melatonin are considered i.e., decreased injury to the lung tissue caused by nicotine, anti-inflammatory and antioxidative effects [34,48]. These studies do not take into account the effects of melatonin through melatonin receptors. In our studies we observed a significant increase in the level of MT2 in smoking NSCLC patients. The analysis of OS indicates that higher expression of MT2 receptor was associated with longer patient survival in the group of smoking NSCLC patients. A multivariate analysis showed that MT2 can be an independent positive prognostic factor in the group of smoking patients. 

Among never smokers with lung cancer, over 65% of patients were diagnosed with AC [49]. SCC mostly develops in smokers. AC and SCC exhibit range of different mutation and they can be used for development new therapeutic strategies [50]. Of note, IHC expression of both melatonin receptors was significantly lower in AC in relation to SCC. This fact was confirmed by the results of our research on tissue material as well as by selected lung cancer cell lines: NCI-H1703 (SCC) and A549 (AC) at protein and mRNA levels. Variances in expression levels related to tumor subtypes may be related with different roles and importance in the tumorigenicity of the cancer cells [51]. The relationship between the expression of both receptors and markers differentiating the histological type of NSCLC was also investigated: p63 (SCC+, AC-) and TTF1 (SCC-, AC+). In the case of MT1, a low positive correlation was obtained with p63 in the whole study group. Interestingly, MT2 correlated positively with p63 and negatively with TTF1 in all studied cases, which suggests the association of the second type of melatonin receptor with the SCC type of NSCLC. Melatonin has been reported to exhibit tumorostatic properties in different tumor models that include tumors of epithelial origin. First reports describing the differences in MT1 and MT2 expression were showed by Slominski et al. in the skin cells. Expression of MT1 and MT2 measured by immunocytochemical studies and RT-PCR showed that melatonin receptors expression was higher in skin stratum granulosum, stratum spinosum than in cells of eccrine sweat gland [52,53]. Histogenesis of SCC of lung cancer is also associated with the transformation of cells of respiratory epithelium which may indicate a greater importance of these receptors in the SCC subtype. This is important due to the possibility of using melatonin in therapy of NSCLC. Initial study of melatonin in the SCC line of cervical origin showed inhibition of cell viability by melatonin [52,53]. Our own observations, as well as other reports, describe a positive correlation between p63 expression with smoking history and overexpression of TTF1 in never smokers [54]. In view of the above facts, MT2 relationship with the smoking status is very likely. Our observations may point to the involvement of MT2 in the pathogenesis of the SCC subtype of NSCLC, especially among smoking patients. Lin et al. hypothesized that betel-quid and tobacco carcinogens might alter MTNR1A promoter activity dependent on the presence of rs2119882, rs13140012, and rs 6553010 polymorphisms. Additionally differential gene expression in ACs versus SCCs may be associated with epigenetic changes. It has been hypothesized that in human cancers, including oral squamous cell carcinoma MTNR1A is a target for epigenetic silencing and that DNA methylation of 5’-CpG islands is a major cause of tumor-suppressor gene inactivity. In these tumors, there was an inverse correlation between MT1 receptor expression and DNA methylation [55]. Interestingly, we also showed the prognostic significance of p63 in the whole NSCLC patient cohort, which is in line with the results of other researchers who showed that high level of p63 expression was an independent prognostic factor for good survival [56]. p63, a marker for squamous differentiation, is a homolog of p53. It can recognize canonical p53 DNA-binding sites and, when overproduced, it can activate p53-responsive target genes [57]. p53 is a protein that plays a key role in promoting the repair of DNA damage as well as in preventing apoptosis. Mutations in p53 are related with advanced grade of NSCLC suggesting a role in tumor progression. Melatonin through melatonin receptor promote the p53 dependent DNA repair which may be important in the early stages of cancer development [58]. 

An important mechanism explaining the melatonin-mediated inhibition of tumor growth is related to the inhibition of the phosphoinositide 3-kinase/protein kinase B (PI3K/AKT) and EGFR signaling pathways in various cancer cell lines [36]. The AKT signaling pathway is upregulated in lung cancer cells and promotes proliferation and apoptosis resistance. The activation of MT1 decrease intracellular level of cAMP, reduce AKT expression and lead to the suppression of cancer cell proliferation [45]. Recently, Steuer et al. noted EGFR overexpression in 39% of AC, and in 58% cases of SCC [37]. This finding seems to be in accordance with our results showing a positive expression of this protein in 28.3% of AC and in 55.9% cases of SCC [59]. Gefitinib and erlotinib are tyrosine kinase inhibitors (TKIs) used in EGFR-directed standard therapies. Mutations in EGFR can influence the response to targeted therapy [36]. Yun et al. suggested that co-treatment of gefitinib with melatonin might effectively down-regulate EGFR phosphorylation and decrease the viability and it could induce apoptosis in H1975 cells with T790M mutation [36]. It is worth mentioning the Spearman’s correlation test showed a low positive significant correlation of MT1 and MT2 with EGFR protein. The survival analysis demonstrated that a high expression of EGFR in the AC subgroup was significantly associated with a poor patient outcome. Previous investigations revealed an association between EGFR expression in NSCLC and a reduced survival and treatment response [37,60]. 

Some studies report that melatonin and its metabolites are significantly lower in NSCLC patients and are decreased after standard chemotherapy [9]. Furthermore, clinical trials by Lissoni et al. showed that immunotherapy and chemotherapy with orally administered melatonin increased the tumor regression rate and prolonged survival time in NSCLC patients [27,61]. Combination of melatonin with interleukin-2 (IL-2) promoted tumor regression and improved 3-year survival in NSCLC patients [61]. Furthermore, some authors report that melatonin increased the efficiency of cytostatics (doxorubicin, cisplatin, etoposide) via an influence on cancer cell proliferation, apoptosis and improved 1-, 2-, 5-year survival in untreated metastatic NSCLC patients [27,30,41,62]. Moreover, in recent studies on invasive ductal carcinoma (IDC) we demonstrated an association between a higher MT1 expression and a favorable prognosis [24]. In the case of NSCLC patients a statistical analysis of OS indicated that a higher expression of MT2 receptor was associated with a longer patient survival. Additional multivariate analysis showed that MT2 can be an independent positive prognostic factor in the whole study cohort. 

Several studies underlined the role of exogenous melatonin in the regulation of smooth muscle tone, lung mucus production, and proinflammatory cytokine levels in human bronchial epithelial cell lines [63,64]. Interestingly, only one paper considered the involvement of melatonin receptors in terms of the mechanism of action of melatonin in lung cancer cells [65]. Those researchers reported that melatonin suppressed the doxorubicin (DOX)-induced premature senescence of A549 lung cancer cells through the direct reactive oxygen species (ROS)-scavenging activity, by protecting mitochondria from genotoxic stress-induced dysfunction. However, an experiment with luzindole did not confirm the participation of melatonin receptors in this mechanism [65]. Participation of the MT2 melatonin receptor in the oncostatic action of melatonin is rarely reported in the literature due to its absence or expression at a low density. Many papers have investigated a role of MT2 in glucose metabolism and its association with the risk of type 2 diabetes [66]. 

Our studies are not free from several limitations. It is a single institutional study including only patients of the Western population. It still need to be validated in a multicenter subgroup of NSCLC to determine the influence of melatonin receptors on patients survival and their role as prognostic and predictive markers.

## 4. Materials and Methods 

### 4.1. Tissue Samples

The tissue material was obtained during surgical resection of lung parenchyma (pneumonectomy, lobectomy) or sublobular resection (segmentectomy, wedge resection) in patients with lung cancer diagnosed between 2007–2017 in the Department of Thoracic Surgery of Wroclaw Medical University, Poland. All study samples were collected before chemotherapy and radiotherapy administration. Immunohistochemical (IHC) studies were conducted using 786 archival paraffin-embedded tissue samples of NSCLC. Histopathological evaluation of hematoxylin-eosin stained slides was used to determine the type and the malignancy grade of the tumors (G) according to the WHO criteria [67]. The paraffin-embedded tissue cases included 120 non-malignant lung tissue (NMLT), 324 squamous cell carcinomas (SCC), 307 adenocarcinomas (AC), 32 adenosquamous carcinomas, 50 large cell carcinomas (LCC), 65 not otherwise specified (NOS) and 8 typical carcinoid tumors. The TNM tumor classification was performed according to the recommendations of The International Association of the Study of Lung Cancer (IASLC) [68]. Table 3 shows the clinico-pathological characteristics of the patients. A total of 574 men (73%) and 212 women (27%) were enrolled in the study. The mean age of the patients was 62.96 ± 8.45 years (21–87 years). Over 80% of the subjects were smokers (n = 665); no information was collected about variation in the smoking status of patients (currently, never, past smoking). The patients were followed up for 50 ± 34.45 months. During this time, 324 (41.2%) patients showed progression and 462 (58.8%) died of the disease. Molecular studies were performed on frozen 62 NSCLC fragments (30 AC and 32 SCC) diagnosed from 2007 to 2017 and 24 NMLT as the control. All studies were conducted with the consent of the Ethics Committee of Wroclaw Medical University (approval: KB-535/2017, date: 8th August 2017).

### 4.2. Construction of Tissue Microarrays (TMA)

The lung TMA was constructed with archival formalin-fixed, paraffin-embedded lung tissue samples. All tumors were reviewed by at least two pathologist-researchers (Piotr Dziegiel, Katarzyna Nowinska). For the construction of the TMA the morphologically representative areas of collected tissues were selected using Pannoramic Midi II histology scanner (3D Histech, Budapest, Hungary) and the Pannoramic Viewer program (3D Histech, RRID:SCR_014424, Budapest, Hungary). At least three core tissue biopsies were punched into 1.5 mm diameter cylinders into new paraffin blocks (recipient paraffin block) using a tissue-arraying instrument (TMA Grand Master equipment; 3D Histech, Budapest, Hungary).

### 4.3. TMA Immunohistochemistry (IHC)

TMA blocks were cut into 4-μm sections. IHC reactions were performed using Dako Autostainer Link48 (Dako, Glostrup, Denmark). Deparaffinization, rehydration and epitope retrieval (97 °C, 20 min) were performed using PT-Link (Dako, Glostrup, Denmark) in EnVisionTM FLEX Target Retrieval Solution High pH (9.0) (Dako/Agilent Technologies, Santa Clara, CA, USA) for antibodies: MT1, p63 and TTF-1, whereas EnVisionTM FLEX Target Retrival Solution Low pH (6.0) (Dako/Agilent Technologies, Glostrup, Denmark/Santa Clara, CA, USA) was used for antibodies to MT2 and EGFR. Endogenous peroxidase was blocked using EnVisionTM FLEX Peroxidase-Blocking Reagent (Dako/Agilent Technologies Glostrup, Denmark/Santa Clara, CA, USA) (5 min). EnVisionTM FLEX (Dako/Agilent Technologies, Glostrup, Denmark/Santa Clara, CA, USA) system was used to visualize IHC reactions performed with the use of polyclonal antibodies: MT1 (rabbit, non-commercial (immunogen: peptide 536), Invitrogen, Carlsbad, CA, USA, RRID:AB_2755008; 1:3200, 20 min, room temperature (RT)), MT2 (rabbit, GenWay Biotech Inc., San Diego, CA, USA, Cat# GWB-15FC18, RRID:AB_10122602; 1:150, 20 min, RT), EGFR (rabbit, Sigma, Munich, Germany; Cat# HPA018530, RRID:AB_1848044; 1:100, 20 min, RT). Morphologically indeterminate NSCLC cases were stained with TTF-1 (mouse, Dako, Glostrup, Denmark/Santa Clara, CA, USA, Cat# IR056, RRID:AB_2755006; dilution: 1:50, 20 min. RT) and p63 (1:300 dilution; Dako, Glostrup, Denmark/Santa Clara, CA, USA, Cat# IR622, RRID:AB_2755007, 20 min. RT) to identify AC and SCC differentiation, respectively. Secondary goat anti-rabbit immunoglobulin antibodies (EnVision/HRP; Dako/Agilent Technologies, Glostrup, Denmark/Santa Clara, CA, USA) were coupled to a dextran core linked to peroxidase. The color reaction was obtained using 3,3’-diaminobenzidine tetrachlorohydrate. Sections were counterstained with hematoxylin (EnVisionTM FLEX Hematoxylin; Dako/Agilent Technologies, Glostrup, Denmark/Santa Clara, CA, USA). The visualization systems were used according to the manufacturer’s instructions. Negative control sections were generated in the absence of a primary antibody.

### 4.4. Cell Lines

For the MTNR1A and MTNR1B mRNA and protein expression studies, two lung cancer cell lines were selected: NCI-H1703 (ATCC, Manassas, VA, USA; Cat# CRL-5889, RRID:CVCL_1490), A549 (ATCC, Cat# CCL-185, RRID:CVCL_0023), and IMR-90 (ATCC, Cat# CRL-7931, RRID:CVCL_0347) as the control. NCI-H1703 was derived from stage I lung SCC. A549 represents AC cell line while IMR-90 is the cell line of human lung fibroblast. NCI-H1703 cells were cultured in RPMI 1640 (Lonza, Basel, Switzerland) supplemented with 10% fetal bovine serum and 2 mM L-glutamine, while A549 and IMR-90 cells were cultured in Dulbecco’s Modified Eagle Medium (DMEM) supplemented with 10% fetal bovine serum and 2 mM L-glutamine for complete media. Cells were grown in 5% CO_2_ in air-humidified incubator at 37 °C.

### 4.5. Evaluation of Immunohistochemical Reactions

Evaluation of individual histological slides was conducted by two independent investigators (Piotr Dziegiel, Katarzyna Nowinska) using the BX-41 light microscope (Olympus, Tokyo, Japan, RRID:SCR_017022). The evaluation and comparison of MT1 and MT2 expression was performed using the semi-quantitative immunoreactive score scale (IRS) of Remmele and Stegner [69]. The scale takes into account the percentage of cells with a noticeable reaction (A) and the intensity of the color reaction (B). The final score represents the product of the two values, ranging from 0 to 12 (A × B). The scoring used in the assessment of EGFR immunostaining was as follows: score 0 = no staining or unspecific staining of tumor cells; score 1 = weak (intensity) and incomplete staining (quality) of more than 10% of tumor cells (quantity); score 2 = moderate and complete staining of more than 10% of tumor cells; score 3 = strong and complete staining of more than 10% of tumor cells [70]. Intensity of TTF-1 and p63 antigen expression in tumor cells was evaluated according to the percentage of positive tumor cells as compared to all tumor cells: 0 pts—no reaction, 1 pt—1–10%, 2 pts—11–25%, 3 pts—26–50%, 4 pts—>50% [23]. For TTF-1 and p63 only nuclear staining was recorded as positive. 

### 4.6. Western Blot

Cell lines NCI-H1703, A549 and IMR-90 were subjected to trypsinization and scored in a hemocytometer. For every western blotting test 5–10 × 10^6^ cells were sampled. After washing in cold phosphate buffered saline (PBS), the cells were subjected to lysis for 20 min in ice with the addition of radioimmunoprecipitation assay buffer (RIPA buffer) (50 mM Tris-Cl pH 8.0, 150 mM NaCl, 0.1% sodium deodecysulfate (SDS), 1% Igepal CA-630 (Sigma, Darmstadt, Germany), 0.5% sodium deoxycholate (Sigma, Darmstadt, Germany), cocktail of protease inhibitors (Sigma, Darmstadt, Germany) and 0.5 mM PMSF (Sigma, Darmstadt, Germany). Protein concentration was measured using the BCA technique (Thermo-Fisher, Waltham, MA, USA) and NanoDrop 1000 spectrophotometer (Thermo-Fisher, Waltham, MA, USA; RRID:SCR_015804). Cell extracts were mixed with sample buffer (250 mM TRIS pH 6.8, 40% glycerol, 20% (*v*/*v*) β-mercaptoethanol, 100 mM DTT, 0.33 mg/mL bromophenol blue, 8% SDS) and then subjected to denaturation for 10 min at 95 °C. Equal amounts of protein (50μg per lane) were separated by electrophoresis in a 10% gel, in the Mini Protean 3 apparatus (BioRad, Marnes-la-Coquette, France). Subsequently, the proteins were electrophoretically transferred to nitrocellulose membrane (GE Healthcare, Whatman, Boston, MA, USA, Protran BA85, 0.45 µm) and sites of non-specific binding were blocked using 4% Bovine serum albumin (BSA) (MT1) in 0.1% TBST buffer (BioRad, Marnes-la-Coquette, Paris, France); 2% milk in 0.1% TBST buffer (BioRad, Marnes-la-Coquette, Paris, France) (MT2). The expression of MT1 was detected using polyclonal MT1-specific antibodies (Invitrogen, Carlsbad, CA, USA), while MT2 was detected with using rabbit polyclonal anti-Melatonin Receptor 1B antibody (Abcam, Cambrige, UK, Cat# ab229544, RRID:AB_2755009). The incubation was conducted for 16 h at 4 °C with delicate shaking in the solution of MT1 antibody diluted 1:4500 in 1% BSA in 0.2% TBST; MT2 antibody diluted 1:400 in 1% milk in 0.1% TBST (Tris-buffered saline (TBS) with Tween 20). The membrane was rinsed 4 times in TBST buffer and subsequently incubated for 1 h in a solution of donkey anti-rabbit antibody, conjugated with peroxidase (1:3000, Jacksons Immunoresearch, Cambridgeshire, UK, Cat# 711-035-152, RRID:AB_10015282). The detection was conducted using a substrate for chemiluminescence (Immun-Star HRP Chemiluminescent Kit, BioRad, Marnes-la-Coquette, Paris, France), and the results were documented for exposure times ranging from 2 s to 30 min in Chemi-Doc XRS Molecular Imager apparatus (Bio-Rad, Marnes-la-Coquette, Paris, France, RRID:SCR_014210). The amount of the applied protein was controlled by staining the total protein on the membrane using Ponceau S (Sigma, Darmstadt, Germany). Relative optical density values of the bands were normalized to reference bands (β-actin) and presented as the means for percentage values of MT1/β-actin and MT2/β-actin optical density ratios. Optical density values of the bands were obtained from three independent experiments.

### 4.7. Immunofluorescence (IF)

NCI-H1703, A549 and IMR-90 cell lines were set up 2 × 10^4^ cells per well Millicell EZ 8-wells glass slide (Merck, Darmstadt, Germany, Cat# PEZGS0816) in appropriate medium overnight. The cells were fixed by 12 min incubation in 4% paraformaldehyde at room temperature (RT). The membranes were permeabilized using 0.2% Triton (10 min/RT). Sites of non-specific binding were blocked using 3% BSA in 0.1% TBST buffer (1 h/RT). The cells were incubated for 1h at RT with primary MT1-specific antibodies, diluted 1:3200 (Invitrogen, Carlsbad, CA, USA) in 1% BSA in PBS and MT2-specific antibodies diluted 1:100 in 1% BSA in PBS (Abcam, Cambridge, UK, Cat# ab203346, RRID:AB_2783824). Subsequently, secondary anti-rabbit antibodies Alexa 568 were applied (1:2000, 1h/RT; Abcam, Cambridge, UK, Cat# ab175470, RRID:AB_2783823). Negative controls were performed with 1% BSA in PBS instead of the specific antibody. The preparations were mounted in a ProLong Gold Antifade Mountant with 4′,6-diamidino-2-phenylindole (DAPI) (ThermoFisher, Waltham, Massachusetts, USA). A Confocal Laser Scanning Microscope Fluoview FV3000 (Olympus, Hamburg, Germany, RRID:SCR_017015) coupled with CellSense software (Olympus, Hamburg, Germany, RRID:SCR_016238) was used for fluorescence. 

### 4.8. Real-Time PCR

Total RNA was isolated from NSCLC, NMLT and cell lines using RNeasy Mini Kit (Qiagen, Hilden, Germany), in line with the manufacturer’s recommendations. Concentration and the quality of the isolated RNA was measured in the NanoDrop1000 (Thermo-Fisher Waltham, Massachusetts, USA, RRID:SCR_015804). Reverse transcription reactions were performed using the High Capacity cDNA Reverse Transcription Kit (Applied Biosystems, Foster City, CA, USA). The reactions were performed in triplicates and evaluated by real-time PCR, using 7500 Fast Real-Time PCR System (Applied Biosystems, Foster City, CA, USA, RRID:SCR_014596), primers and probes of TaqMan system (Applied Biosystems, Foster City, CA, USA). The primers and probes used in the reactions involved MTNR1A Hs00195567_m1 for *MTNR1A*, MTNR1B Hs04981113_s1 for *MTNR1B* and ACTB Hs99999903_m1 for *β-actin* (Applied Biosystems, Foster City, CA, USA). Thermal cycling conditions were as follows: polymerase activation at 50 °C for 2 min, preliminary denaturation at 94 °C for 10 min, denaturation at 94 °C for 15 s, annealing of primers and probes and synthesis at 60 °C for 1 min, for 40 cycles The results were standardized in relation to the expression of the reference gene of β-actin. Relative expression (RQ) of MTNR1A and MTNR1B mRNA was calculated using ΔΔCt method.

### 4.9. Statistical Analysis

The results were subjected to statistical analysis using Prism 5.0 software (GraphPad, San Diego, CA, USA, RRID:SCR_002798) and Statistica 13 (StatSoft, Cracow, Poland, RRID:SCR_014213). Clinical and pathological data of NSCLC cases were compared to the expression levels of MT1 and MT2. The correlation analysis was performed using the Spearman’s rank correlation coefficient. Kruskal-Wallis and Mann-Whitney tests with post-hoc Dunn’s or the Bonferroni Multiple Comparison Test were used to compare the groups of data that did not meet the assumptions of the parametric test. Survival analysis was performed using the Kaplan-Meier method. Multivariate analysis was performed using the Cox Proportional Hazards Regression model. For each variable, the hazard ratio (HR) and 95% confidence interval (CI) were determined. The results were considered statistically significant at p < 0.05 in all the analyses.

## 5. Conclusions

Although both MT1 and MT2 receptors are expressed in NSCLC cases, it seems that the MT2 receptor predominates in this type of tumor. It may be due to different mechanisms underlying carcinogenesis and the biology of this type of tumor cells. The observed elevated expression of melatonin receptors both at the protein and mRNA levels in the SCC subtype suggests their potential role in lung cancer biology. Our results were obtained on tissue material from large group of NSCLC patients with well characterized clinicopathological data and a follow-up of over 5 years. The IHC analysis of MT1 and MT2 showed the significant relations between the melatonin receptors and the patient clinicopathological data (e.g., clinical cancer stage, tumor size or the presence of lymph node metastases). Our results showed for the first time a favorable prognostic significance of MT2 for overall survival. This observations may point to the potential role of melatonin receptors in the pathogenesis and progression of NSCLC. Future studies, especially in vitro experiments, may help to elucidate the role of melatonin receptors in lung cancer biology and confirm its prognostic and predictive value.

## Figures and Tables

**Table 1:** Expression (immunohistochemistry, IHC) of the analysed antigens in total, adenocarcinoma (AC) and squamous cell carcinoma (SCC).

Parameter	Histology	Mean (SD)	Median
**MT1**	**Total**	6.2 (3.4)	6
	**AC**	6 (3.6)	
	**SCC**	6.8 (3.3)	
**MT2**	**Total**	4.1 (3.1)	3
	**AC**	2.9 (2.4)	
	**SCC**	5.5 (3.2)	
**EGFR**	**Total**	0.7 (1)	0
	**AC**	0.5 (0.9)	
	**SCC**	1 (1)	
**p63**	**Total**	2.1 (1.7)	2
	**AC**	1 (1.2)	
	**SCC**	3.4 (1.2)	
**TTF-1**	**Total**	1.8 (1.6)	1
	**AC**	2.9 (1.4)	
	**SCC**	0.9 (1.1)	

SD, standard deviation; MT1, MT2, melatonin receptors; EGFR, epidermal growth factor receptor; AC, adenocarcinoma; SCC, squamous cell carcinoma; TTF-1, thyroid transcription factor-1.

**Table 2:** Survival analysis of A) the whole cohort, B) non-smoking and smoking patients, C) adenocarcinoma (AC) and squamous cell carcinoma (SCC). Cox proportional hazard regression.

**A**								
**Overall Survival (OS)**								
**Clinico-pathological Parameter**			**All**					
	**Univariate**	**Multivariate**						
	***p*-value**	**HR**	**95% HR CI**	***p*** **-value**				
Gender (female vs male)	**0.0003**	1.4899	1.1794–1.8823	**0.0008**				
Age (<60 vs ≥60)	**0.0018**	1.4805	1.2176–1.8002	**0.00008**				
Tumor size (pT1 vs pT2–T4)	**<0.0001**	1.401	1.1054–1.8243	**0.0061**				
Lymph nodes (pN- vs pN+)	**<0.0001**	1.5495	1.2324–1.9483	**0.0002**				
Stage (I vs II-IV)	**<0.0001**	1.3223	1.1138–1.7246	**0.0039**				
Smoking (no vs yes)	**0.0365**	1.1872	0.893–1.5783	0.2274				
Melatonin receptor MT1 (low vs high)	0.3927	-	-	-				
Melatonin receptor MT2 (low vs high)	**0.0049**	0.8069	0.6628–0.9823	**0.0324**				
p63 (<25% vs ≥25%)	**0.0449**	0.7873	0.646–0.9596	**0.0018**				
**B**								
	**Overall Survival (OS)**							
**Clinico-pathological Parameter**	**Non-Smoking Patients**				**Smoking Patients**			
	**Univariate**	**Multivariate**			**Univariate**	**Multivariate**		
	***p*-value**	**HR**	**95%** **HR CI**	***p*-value**	***p*-value**	**HR**	**95%** **HR CI**	***p*-value**
Gender (female vs male)	**0.03**	1.62	0.925–2.8427	0.0914	**0.0199**	1.3478	1.0454–1.7376	**0.0212**
Age (<60 vs ≥60)	0.2212	-	-	-	**0.0113**	1.4436	1.1704–1.7805	**0.0006**
Tumor size (pT1 vs pT2–T4)	0.0888	-	-	-	**<0.0001**	1.4534	1.1085–1.9058	**0.0068**
Lymph nodes (pN- vs pN+)	0.0942	-	-	-	**<0.0001**	1.6264	1.2732–2.0777	**0.0001**
Stage (I vs II–IV)	**0.0024**	2.2151	1.2262–4.0016	**0.0084**	**<0.0001**	1.2265	0.9227–1.6303	0.1597
Smoking (no vs yes)	-	-	-	-	-	-	-	-
Melatonin receptor MT1 (low vs high)	0.3062	-	-	-	0.5774	-	-	-
Melatonin receptor MT2 (low vs high)	0.2244	-	-	-	**0.0043**	0.7524	0.6166–0.9181	**0.0051**
p63 (<25% vs ≥25%)	0.1854	-	-	-	0.0631	-	-	-
**C**								
**Overall Survival (OS)**								
**Clinico-pathological Parameter**	**Adenocarcinoma (AC)**				**Squamous Cell Carcinoma (SCC)**			
	**Univariate**	**Multivariate**			**Univariate**	**Multivariate**		
	***p*-value**	**HR**	**95%** **HR C**	***p*-value**	***p*-value**	**HR**	**95%** **HR C**	***p*-value**
Gender (female vs male)	**0.0018**	1.608	1.1378–2.2724	**0.0071**	0.2972	-	-	-
Tumor size (pT1 vs pT2–T4)	**0.0093**	1.5116	1.0256–2.2281	**0.0368**	**0.0029**	1.5846	1.0448–2.4032	**0.0303**
Lymph nodes (pN- vs pN+)	**<0.0001**	1.8762	1.2851–2.7393	**0.001**	**0.0199**	1.301	0.9127–1.9094	0.1402
Malignancy grade (G1 vs G2-G3)	0.5698	-	-	-	**0.0364**	1.4314	0.9783–2.094	0.0647
Stage (I vs II–IV)	**<0.0001**	1.2001	0.7749–1.8586	0.41368	**0.0038**	1.1052	0.721–1.6942	0.6461
Smoking (no vs yes)	**0.0248**	1.4481	0.9169–2.2968	0.1112	0.5221	-	-	-
Melatonin receptor MT1 (low vs high)	0.1514	-	-	-	0.5196	-	-	-
Melatonin receptor MT2 (low vs high)	0.2937	-	-	-	0.077	-	-	-
EGFR (low vs high)	**0.0357**	1.4349	1.0523–1.9565	**0.0225**	0.8002	-	-	-

A: Significant *p*-values are given in bold. HR, hazard ratio; CI, confidence interval; MT1, MT2 melatonin receptors; pT, tumor size; pN, lymph nodes status. B: Significant *p*-values are given in bold. HR, hazard ratio; CI, confidence interval; MT1, MT2 melatonin receptors; pT, tumor size; pN, lymph nodes status.C: Significant *p*-values are given in bold. HR, hazard ratio; CI, confidence interval; MT1, MT2 melatonin receptors; pT, nodes tumor size; pN, lymph nodes status; EGFR, epidermal growth factor receptor.

**Table 3:** Clinical and pathological characteristics of 786 patients with non-small cell lung carcinoma (NSCLC).

	Total		Adenocarcinoma (AC)		Squamous Cell Carcinoma (SCC)	
Characteristics	N = 786	%	N = 307	%	N = 324	%
**Age**						
≤50	38	4.8	20	6.5	9	2.8
>50	749	95.2	287	93.5	315	97.2
**Gender**						
Male	574	73	199	64.8	264	81.5
Female	212	27	108	35.2	60	18.5
**Tumor grade**						
G1	43	5.5	28	9.1	10	3.1
G2	572	72.8	204	66.4	258	79.6
G3	138	17.6	72	23.5	53	16.4
G4	0	0	0	0	0	0
N/A	33	4.2	3	1	3	0.9
**Tumor size**						
pT1	208	26.5	78	25.4	86	26.5
pT2	341	43.4	175	57	141	43.5
pT3	156	19.8	51	16.6	66	20.4
pT4	81	10.3	3	1	31	9.6
**Lymph nodes**						
pN0	521	66.3	193	62.9	215	66.4
pN1	144	18.3	46	15	77	23.7
pN2	121	15.4	68	22.1	32	9.9
**Metastases**						
M0	780	99.2	303	98.7	323	99.7
M1	6	0.8	4	1.3	1	0.3
**Stage**						
I	295	37.5	117	38.1	121	37.3
II	263	33.5	86	28	124	38.3
III	221	28.1	101	32.9	78	24.1
IV	7	0.9	3	1	1	0.3
**Smoking status**						
Neg.	121	15.4	53	17.3	27	8.3
Pos.	665	84.6	254	82.7	297	91.7
**Living in the city**						
Neg.	731	93	288	93.8	299	92.3
Pos.	55	7	19	6.2	25	7.7
**Distal Metastases**						
Absent	786	100	307	100	324	100
Present	0	0	0	0	0	0
**p63**						
Neg.	205	26.1	134	43.6	18	5.6
Pos.	571	72.6	169	55.1	304	93.8
N/A	10	1.3	4	1.3	2	0.6
**TTF-1**						
Neg.	220	28	30	9.8	134	41.4
Pos.	556	70.7	271	88.2	187	57.7
N/A	10	1.3	6	2	3	0.9
**EGFR**						
Neg.	469	59.7	220	71.7	143	44.1
Pos.	317	40.3	87	28.3	181	55.9

TTF-1, Thyroid transcription factor-1; EGFR, epithelial growth factor; N/A, non-available.
